# Computational Analysis of Mechanical Performance for Composite Polymer Biodegradable Stents

**DOI:** 10.3390/ma14206016

**Published:** 2021-10-13

**Authors:** Žiga Donik, Branko Nečemer, Matej Vesenjak, Srečko Glodež, Janez Kramberger

**Affiliations:** Faculty of Mechanical Engineering, University of Maribor, Smetanova ul. 17, 2000 Maribor, Slovenia; ziga.donik@um.si (Ž.D.); branko.necemer@um.si (B.N.); matej.vesenjak@um.si (M.V.); srecko.glodez@um.si (S.G.)

**Keywords:** vascular stent, bioresorbable polymer, composite, finite element analysis, mechanical performance

## Abstract

Bioresorbable stents (BRS) represent the latest generation of vascular scaffolds used for minimally invasive interventions. They aim to overcome the shortcomings of established bare-metal stents (BMS) and drug-eluting stents (DES). Recent advances in the field of bioprinting offer the possibility of combining biodegradable polymers to produce a composite BRS. Evaluation of the mechanical performance of the novel composite BRS is the focus of this study, based on the idea that they are a promising solution to improve the strength and flexibility performance of single material BRS. Finite element analysis of stent crimping and expansion was performed. Polylactic acid (PLA) and polycaprolactone (PCL) formed a composite stent divided into four layers, resulting in sixteen unique combinations. A comparison of the mechanical performance of the different composite configurations was performed. The resulting stresses, strains, elastic recoil, and foreshortening were evaluated and compared to existing experimental results. Similar behaviour was observed for material configurations that included at least one PLA layer. A pure PCL stent showed significant elastic recoil and less shortening compared to PLA and composite structures. The volumetric ratio of the materials was found to have a more significant effect on recoil and foreshortening than the arrangement of the material layers. Composite BRS offer the possibility of customising the mechanical behaviour of scaffolds. They also have the potential to support the fabrication of personalised or plaque-specific stents.

## 1. Introduction

Nowadays, cardiovascular diseases are among the most dangerous diseases in the world [1]. Stenting interventions are used to restore normal blood flow through partially or completely blocked blood vessels. Coronary stents are usually tiny wire mesh tubes used to open arteries that have become blocked over time due to the accumulation of fat, cholesterol, or other substances. Generally, coronary stents are considered permanent implants. The treatment involves the endovascular insertion of an expandable, cylindrical cardiovascular device into the vessel. The stent is contracted to a small diameter, placed over an angioplasty balloon catheter, and pushed into the area of blockage. When the balloon is inflated, the stent expands, plastically deforms, locks in place, and forms a scaffold that keeps the artery open. Inserting stents into narrowed blood vessels to restore normal blood flow is a less invasive method of treating cardiovascular disease [2]. Currently available balloon-expandable stents are made of medical-grade stainless steel (316L), nickel-titanium alloy (NiTi), or cobalt-chromium alloy (CoCr). The latest high-performance coronary artery stents are made of improved metal alloys, often with a drug-eluting outer coating. These drug-eluting stents (DES) offer better clinical performance than earlier technologies. However, permanent stents are a chronic irritant to the host; they interfere with future cardiac interventions and do not conform to the natural behaviour of the vessel [3].

Bioresorbable stents (BRS) have been introduced to overcome the limitations of permanent stents and offer significant advantages, such as complete bioresorption and mechanical flexibility [4,5,6,7]. Bioresorbable stents are usually made of biodegradable polymers or magnesium alloys [8,9,10]. However, due to the relatively weak mechanical properties of polymers compared to metals, polymeric stents usually have poorer mechanical performance [11,12]. On the other hand, advanced manufacturing technologies such as laser cutting [13,14] and 3D printing [15,16,17,18] can be used to fabricate polymer stents. BRS has become an important topic, and many researchers have contributed to its research in recent years. A number of studies have investigated the effect of various geometric parameters on different performance outcomes of stents. Some of the representative results are summarised below.

Schiavone et al. [19] compared the mechanical performance of metallic (Xience) and bioresorbable polymeric (Elixir) stents during the crimping and deployment process. They found that polymeric stents exert less stress on the arterial system due to the smaller discrepancy between the properties of the polymers and the arterial tissue, which could be clinically beneficial. Wang et al. [20] investigated the mechanical properties of a poly-L-lactic acid (PLLA) stent based on the 3D finite element method (FEM) and experimental verification. The deformation of the stent and the distribution of stress and strain were analysed in several deformation steps. Migliavacca et al. [21,22] applied the FEM to understand the effects of different geometric parameters (thickness, metal to arterial surface ratio, longitudinal and radial cut lengths) of a typical diamond-shaped coronary stent on its mechanical performance. Etave et al. [23], Pant et al. [24], Britto et al. [25] and Wei et al. [26] proposed several stent designs with different geometry by varying the shape of the circumferential rings and the links and investigated how to improve the design. Liu et al. [27,28] demonstrated the feasibility of using shape memory polymers (SMPs) for vascular stent design. In particular, the less invasive and stable expansion performance of SMP stents was confirmed by intensive modelling and simulation. In their study, Yu et al. [29] provided a comprehensive overview of the significant potential of mechanical metamaterials and the upcoming challenges in the research field. Khosravi et al. [30] showed that the unwanted deformation of the stent can be reduced by using functionally graded materials (FGM). Their results showed that the material parameters have a crucial influence on the deformation of the stent so that the FGM parameters can be adjusted with respect to the goal of biomechanical optimisation.

As mentioned earlier, most realistic computational studies focused on metallic stents [31,32,33,34]. The computational analysis of realistic bioresorbable polymer stents is very limited. Cabrera et al. [35] chose a commercially available 3D printed polymer. Crush and crimp tests were performed to validate the results predicted by the computational model. Finally, the degradability of the polymer was evaluated by accelerated hydrolysis. In their study, Qiu et al. [36,37] modelled stent crimping and expansion of four commercial bioresorbable polymer stents (i.e., Absorb, Elixir, Igaki-Tamai, and RevaMedical stent) using the FEM. The results showed that these four stents exhibited different mechanical performances during the crimping and expansion process due to their individual designs. The time-dependent mechanical behaviour and performance of bioresorbable polymer stents have rarely been investigated by computational simulations [38,39]. Thus, there is a large gap in the study of the mechanical behaviour of polymer stents with different designs. 

In this study, a novel bioresorbable composite polymer stent is analysed, based on the idea that composite stents are a promising solution to meet the stringent BRS requirements [40]. Numerical analysis results are also compared to available experimental results of layered PLA-PCL stent expansion [40]. FEM is used to investigate the mechanical properties of the proposed composite polymer vascular stent, including the process of radial crimping and expansion. Different configurations of composite materials were investigated for a single geometry of the stent. The mechanical response of the stent was observed and quantified using metrics such as foreshortening and radial elastic recoil. In addition, stresses and plastic strains were analysed to evaluate the effects of residual stresses resulting from crimping and expansion on the mechanical properties of the proposed bioresorbable polymer stent.

## 2. Materials and Methods

### 2.1. Modelling and Simulation

**Figure 1:**
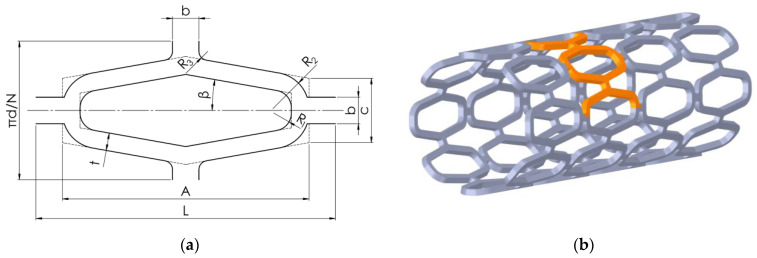
Stent shape: (**a**) Unit cell shape and dimensions; (**b**) full stent with highlighted area for simulation (orange).

The desired stent shape was first modelled using 3D CAD software Solidworks (Dassault Systèmes, Release 2021, Vélizy-Villacoublay, France). The unit cell of the stent is shown in Figure 1a, while its dimensions can be seen in Table 1. The shape of the stent visible in Figure 1b allows the use of symmetry in the numerical model. Therefore, only a part of the stent can be used for the quasi-static simulation, applying appropriate symmetry boundary conditions. The part used for numerical analysis is highlighted in orange in Figure 1b.

**Figure 2:**
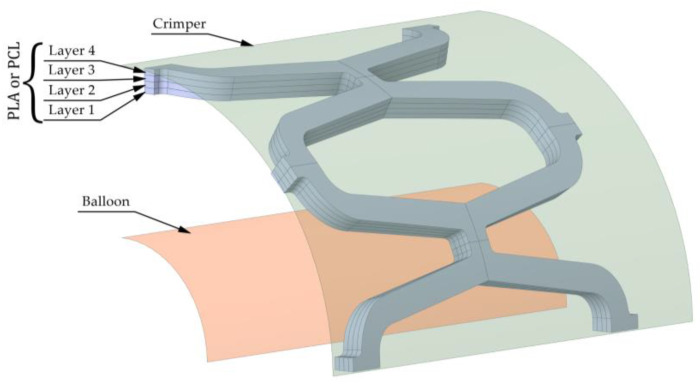
Stent geometry for numerical analysis with balloon and crimper.

A crimper and an expandable balloon were modelled as surfaces to load the stent. The stent has to be compressed to a smaller diameter with a crimper to ensure it can fit through the blood vessel and be placed in the correct spot. The balloon is then inflated to expand the stent. Both the crimper and the balloon can be seen in Figure 2. The initial diameters of the crimper and expandable balloon are 4.5 mm and 1.8 mm, respectively.

The stent was divided into four layers in the radial direction to study the effects of different material combinations on its mechanical properties. The impact of layer configuration on the mechanical properties of the structure was also investigated (i.e., if there was a significant difference in the mechanical response of a structure made of the same number of layers from each material when the layers were variously placed). Four layers were chosen to limit the number of resulting configurations and to be able to reproduce a configuration where half of a stent was made from one, and half from another material for comparison with available experimental results (stent made of two layers). Two layers are not sufficient to compare different configurations of layer placement, and more than four layers would greatly increase the number of possible configurations while most likely not improving outcomes. Two different materials were used for the four different layers, resulting in sixteen unique stent compositions. The materials that made up the 3D-printed composite stent were intended to have similar manufacturing properties while having different material properties to meet different requirements. Two bioresorbable polymeric materials used for the composite stent were polycaprolactone (PCL) and polylactic acid (PLA). The more rigid PLA would provide support, while PCL would provide sufficient flexibility to conform to the vessel wall. Biodegradation is outside the scope of this article, but PCL degrades more slowly than PLA. Therefore, PLA would provide additional support in the early stages of blood vessel remodelling and be resorbed later to provide more flexibility in the late stages when arterial remodelling is complete. Ideally, PCL would decompose when the vessel is completely healed. The elasto-plastic material properties of the stent materials used in the numerical simulations are listed in Table 2. The material combinations in the stents are indicated by four numbers (e.g., 1010), where 0 and 1 represent PLA and PCL, respectively. The numbers represent the layers in order, as shown in Figure 2, where the first number represents the innermost layer (layer 1), and the last number represents the outermost layer (layer 4). For the balloon, a hyperelastic material is considered with a two-parameter material model Mooney−Rivlin (*C*_10_ = 1.069 MPa; *C*_01_ = 0.7109 MPa) adopted from Ju et al. [34]. For the steel crimper, a linear elastic material model with Young’s modulus of 210 GPa and Poisson’s ratio of 0.3 was used. The thickness of the balloon and the crimper is 0.1 mm and 1 mm, respectively.

**Figure 3:**
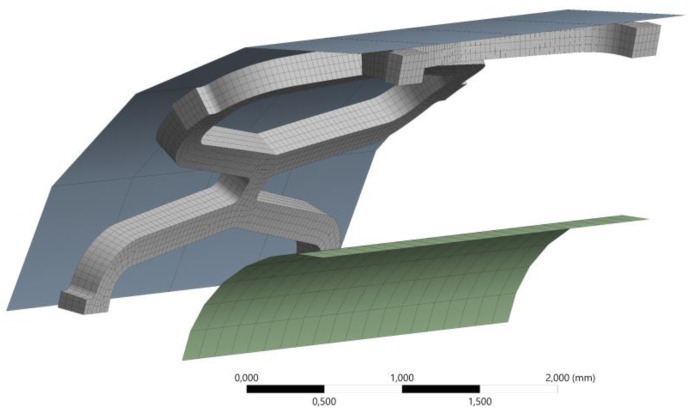
Finite element mesh for numerical analysis.

A solid 3D hexahedral finite element mesh was created for the stent (20 node hexahedral elements), while a shell mesh was used for the crimper and balloon (4 node structural shell). A mesh convergence analysis was performed. The properties of the finite element mesh are summarised in Table 3, and the resulting mesh is shown in Figure 3. The contact area of the crimper usually consists of 8–16 planar surfaces. A coarse mesh on a circular geometry is designed to replicate the crimper, generating 4 elements per 90°, as shown in Figure 3.

**Figure 4:**
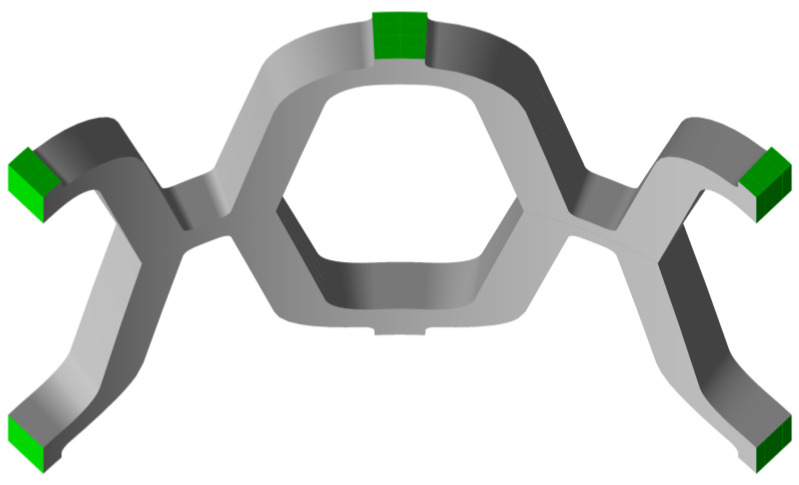
Boundary condition surfaces on the stent (green).

To satisfy the symmetry constraints and to restrict movement, a frictionless boundary condition was prescribed on three sides of the stent, as shown in Figure 4. The contacts between the stent and the crimper, the stent and the balloon, and the stent itself were defined as frictionless. The contact between the struts of the stent is required for the compression phase during crimping. The layers are merged together to form a single part with merged nodes; therefore, no contact definition is required on the layer/layer interface. A cylindrical coordinate system was created to facilitate loading in the radial direction. The loading of the stent was performed by controlling the displacement of the crimper and the balloon. Four simulation steps were required to simulate the crimping and expansion process of the stent. In the first step, the crimper was displaced to compress the stent to an internal diameter, *d*, of 2 mm. In the second step, the crimper was removed so that the stent had enough room to spring back. In the third step, the balloon was inflated by moving the balloon surface to expand the stent to an internal diameter, *d*, of 8 mm. In the final step, the balloon was released to allow elastic recoil of the stent. A nonlinear analysis approach was performed using Ansys^®^ Academic Research Mechanical FEA software (ANSYS, Inc., Release 21.2, Canonsburg, PA, USA).

### 2.2. Radial Elastic Recoil and Foreshortening

**Figure 5:**
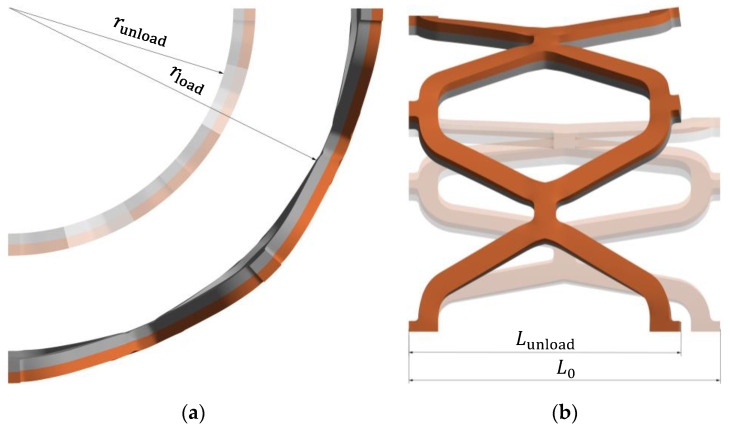
Spring-back effect: (**a**) radial elastic recoil; (**b**) foreshortening.

Elastic recoil is determined by the deformation behaviour of the stent material and the specific stent design. The radial elastic recoil of the stent, *ER*, can be calculated according to ASTM F2079-09 [42]:

(1) $$ER=\frac{r_{load}-r_{unload}}{r_{load}}\cdot 100\%,$$

where $r_{unload}$ is the radius of the stent once the balloon is removed, and $r_{load}$ is the radius of the stent when the balloon is fully inflated. The visual representation is shown in Figure 5a. After insertion of the stent, sufficient displacement in the radial direction is required to ensure adequate lumen area to allow free blood flow through the vessel. A small amount of recoil is necessary to maintain the vessel at the desired diameter and to avoid overstretching the arteries, which can lead to neointimal hyperplasia. The external pressure on the stent caused by the artery is not considered in this study. Therefore, a higher radial elastic recoil can be expected after actual stent deployment in the blood vessel. Nevertheless, this comparative study provides insight into the mechanical behaviour of composite stents.

Foreshortening refers to the difference between the stent’s initial and final length. Stent foreshortening, *f*, can be calculated as:

(2) $$f=\frac{L_{0}-L_{unload}}{L_{0}}\cdot 100\%,$$

where $L_{unload}$ is the length of the stent once the balloon is removed, and $L_{0}$ is the initial length of the stent before inflating the balloon. The dimensions can be seen in Figure 5b. Stent foreshortening can cause damage on the arterial walls during deployment, contributing to neointimal hyperplasia. Therefore, minimal foreshortening is desired.

## 3. Results

The numerical simulation results include stresses, strains, displacements, radial elastic recoil and stent foreshortening. Since this was a comparative study, the focus was on the comparison between different material configurations. Due to some simplifications, absolute result values are not given for evaluation and direct comparison with experimental data.

### 3.1. Equivalent Stress

**Figure 6:**
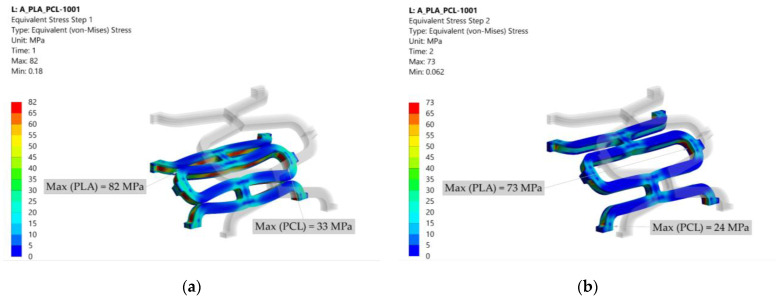

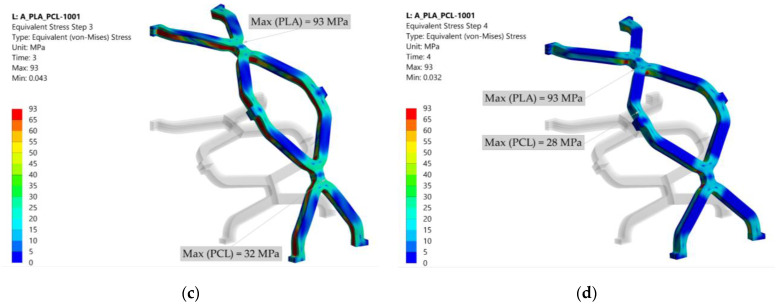
Equivalent (von Mises) stress results in MPa for configuration 1001 (deformation scale factor: 1): (**a**) stress after crimping; (**b**) residual stress after crimper removal; (**c**) stress after expansion; (**d**) residual stress after balloon removal.

The stresses resulting from crimping and expansion depend on the material composition of the stent. The stresses are displacement induced; therefore, lower stresses are expected in the material with the lower modulus of elasticity (i.e., PCL). The results of the equivalent stresses (von Mises) at the end of each simulation step for configuration 1001 are shown in Figure 6. Equivalent stresses after crimping are shown in Figure 6a. The highest stress in PLA occurs at the contact between the struts, while the highest stress in PCL occurs at the unit cell radius. The yield stress (65 MPa for PLA and 25 MPa for PCL) is exceeded, and plastic deformation occurs in the red and green regions for PLA and PCL, respectively. After removing the crimper, the stent may expand to a certain extent (recoil). Return to the initial state is not possible due to irreversible deformation in some areas by crimping. As a result, some residual stresses remain, which can be seen in Figure 6b. These stresses are still above the yield stress for PLA, while they fall into the elastic region for PCL. Stress concentrations occur at the radii of the unit cell for both PLA and PCL since there is no contact between the struts. The highest stresses occur during the expansion of the stent, as shown in Figure 6c. Stress concentrations occur at the intersection of the unit cell with the connecting strut bridging the unit cells and exceed the yield stress for both PLA and PCL. Therefore, these areas are subject to additional plastic deformation. After stent expansion, a slight decrease of stent diameter may be observed when the balloon is removed. The spring back depends on the material composition of the stent and the degree of plastic deformation. High residual stresses can be seen in Figure 6d.

**Figure 7:**
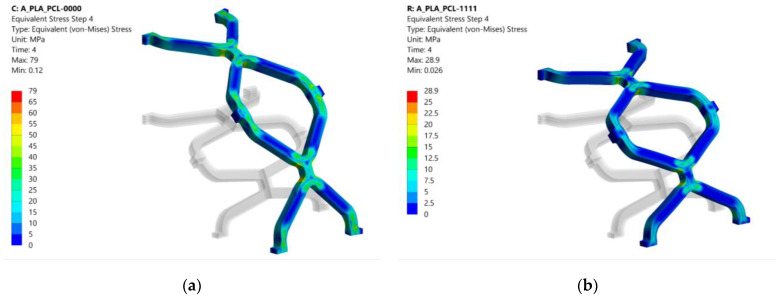
Equivalent (von Mises) stress results in MPa after the last numerical analysis step for configurations 0000 and 1111 (deformation scale factor: 1): (**a**) residual stress after balloon removal for configuration 0000; (**b**) residual stress after balloon removal for configuration 1111.

Similar stress distribution was observed in other composite combinations where the different material layers were separated by a clear line. The residual stresses in PLA-only (0000) and PCL-only (1111) stents after the last simulation step (after balloon inflation and removal) are shown in Figure 7 for comparison with the composite structures. In the PLA stent, the stresses appear to be slightly more evenly distributed across the structure than in the composite stent. In addition, fewer stress concentrations are seen in the PLA-only (0000) and PCL-only (1111) stents than in the composite stent (1001). On the other hand, it is immediately apparent that the pure PCL stent experiences greater elastic recoil due to material flexibility.

The stress analysis shows that the structure is subjected to stresses that significantly exceed the yield stress. Therefore, the structure would most likely fracture under the prescribed load. Nevertheless, the comparison between different material configurations may be valid. Changing the shape of the stent would likely reduce stress concentrations. Changing the geometry could be most effectively achieved by shape or topology optimisation. Further reduction of stress concentrations could possibly be achieved by a different material arrangement of the composite stent (e.g., different combinations of materials at the strut intersections instead of layering the stent).

### 3.2. Equivalent Plastic Strain

**Figure 8:**
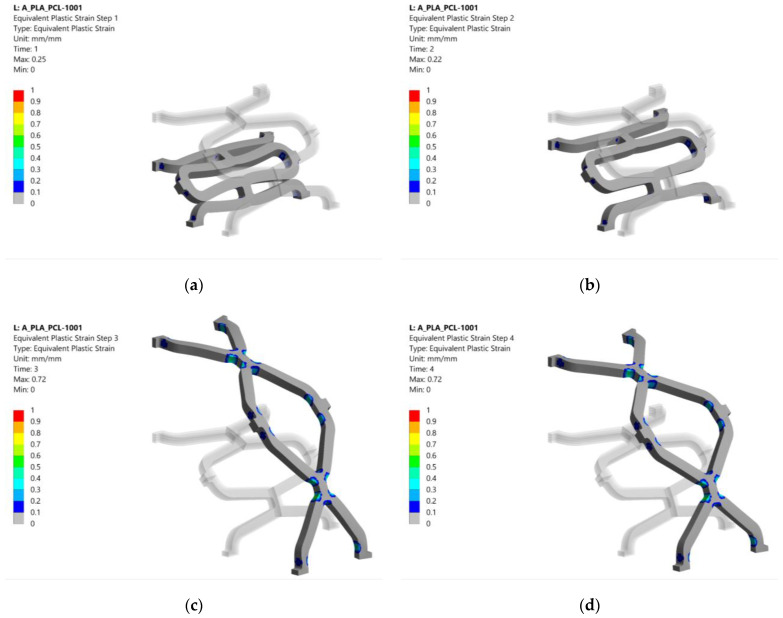
Equivalent plastic strain results for configuration 1001 (deformation scale factor: 1): (**a**) plastic strain after crimping; (**b**) plastic strain after crimper removal; (**c**) plastic strain after expansion; (**d**) plastic strain after balloon removal.

The equivalent plastic strain in configuration 1001 after each analysis step is shown in Figure 8. Equivalent plastic strains after crimping are shown in Figure 8a. Plastification occurs at the radii of the strut intersections, especially at the PLA layers that coincide with the highly stressed regions. The plastic strain remained almost the same after the crimper was released, as shown in Figure 8b. The highest plastic strains were caused during the expansion of the stent. Again, they were located near the strut intersections visible in Figure 8c. After deflation of the balloon, the stent retained most of its deformed shape from the previous step. The remaining plastic strain after removal of the balloon can be seen in Figure 8d.

**Figure 9:**
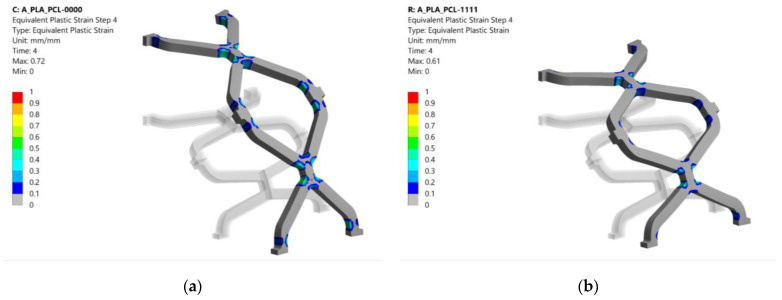
Equivalent plastic strain results after the last numerical analysis step for configurations 0000 and 1111 (deformation scale factor: 1): (**a**) plastic strain after balloon removal for configuration 0000; (**b**) plastic strain after balloon removal for configuration 1111.

The equivalent plastic strains after the last simulation step (balloon deflation) for pure PLA (0000) and PCL (1111) stents are shown in Figure 9 for comparison with the composite stent in Figure 8. Configuration 0000 had a slightly larger plastic area than the other configurations. In contrast, configuration 1111 had an 11% lower equivalent plastic strain, although the area affected was virtually the same as that of the composite stent (1001). This is due to the higher compliance of PCL (lower Young’s modulus, *E*), which results in lower stresses when loaded with the same displacement compared to PLA. Lower stresses in PCL exceed the yield stress less than in PLA, resulting in lower equivalent plastic strain.

The results of the equivalent plastic strain after the last simulation step are summarised in Table 4 for all material combinations. The plastic strain ranged from 0.71 to 0.74 for composite stents with at least two PLA layers, while lower values were observed for configurations with higher PCL content. Configurations with three PCL layers exhibited an equivalent plastic strain of 0.63 to 0.69. For these configurations, the plastic strain increased as the diameter of the PLA layer increases (e.g., highest value when the PLA is on layer 4). The lowest equivalent plastic strain was observed for configuration 1111, as also shown in Figure 8b. Higher plastic strain is desired to decrease the elastic recoil and ensure sufficient luminal area for unobstructed blood flow. Plastic deformation would ideally be present in a larger portion of the structure and be more equally distributed. The distribution could be improved with structural optimisation.

### 3.3. Radial Elastic Recoil and Foreshortening

**Figure 10:**
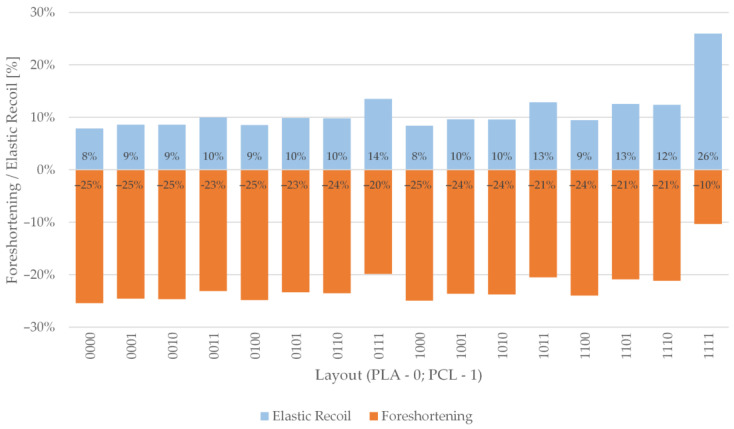
Radial elastic recoil and foreshortening results.

Foreshortening and radial recoil results were calculated during postprocessing in Ansys^®^ Academic Research Mechanical, Release 21.2 FEA code using user-defined results. The initial nodal coordinates and nodal displacements at the desired simulation step were used to calculate foreshortening and radial recoil according to Equations (1) and (2) in Section 2.2. Figure 10 shows the results of radial elastic recoil and foreshortening results for all material combinations. It was immediately apparent that a single configuration stood out from the others. Configuration 1111, a stent composed only of PCL, showed the highest elastic recoil (26%) and the lowest foreshortening (−10%). This behaviour was expected due to the higher flexibility of PCL compared to PLA. The radial elastic recoil for other configurations ranged from 8 to 14%, while the values for shortening ranged from −20 to −25%. This shows that adding only a single PLA layer to a PCL stent has a significant impact on the stiffness of the stent.

The configurations with three PLA layers and a single PCL layer showed similar behaviour with approximately the same foreshortening of −25%. The only difference was seen in the elastic recoil, which was 8% for the configuration 1000 and 9% for the others. Configuration 1000 contained the lowest volume ratio of PCL; therefore, it behaved most like configuration 0000, for which the same results were obtained (*ER* = 8%; *f* = −25%). Similar observations apply to the configurations with the same number of PCL and PLA layers. Composite stents with two PCL and two PLA layers had elastic recoil values between 9 and 10%, while foreshortening was between −23 and −24%. Stents with three PCL and one PLA layer had elastic recoil values between 12 and 14%, while foreshortening was between −20 and −21%.

The results of elastic recovery and foreshortening show that the arrangement of the layers is insignificant for materials with only slight differences in different configurations consisting of the same number of layers from each material. The volumetric ratio between the materials appears to have a greater effect on mechanical performance than the layer arrangement.

## 4. Discussion

The results showed the similar behaviour of material combinations with the same number of layers per material, which was also observed in an experimental study by Guerra et al. [40]. This accounts for stresses, strains, elastic recoil, and foreshortening. Simulation elastic recoil results were comparable to the experimentally obtained data for configurations 1111, 0011 and 1100 performed by Guerra et al. [40] despite slight differences in stent cell shape. The importance of the layer arrangement only becomes apparent when considering the plastic strain for configurations with three PCL layers and one PLA layer. Moving the PLA towards the outer layer (layer 4) increased the resulting plastic strain after the last simulation step (deflation of the balloon). Higher plastic strain is desired to decrease the elastic recoil and ensure sufficient luminal area for unobstructed blood flow. The volume ratio of the materials seems to have a slightly more prominent effect on the mechanical performance than the material arrangement. From the elastic recoil and foreshortening results, the PCL-only stent exhibited the highest recoil and the lowest foreshortening. After deployment, the PCL stent may not provide enough lumen area for unobstructed blood flow due to the high recoil. The radial recoil and radial stiffness could be controlled by using a stiffer material such as PLA or an improved (optimised) stent shape. Instead of dividing a stent into layers, strut junctions and struts could be made from different materials.

Furthermore, the mechanical properties of an atherosclerotic lesion may differ drastically within only a few millimetres along the artery. Fatty deposits exhibit greater compliance, whereas calcium-containing plaque may exhibit considerable stiffness. This requires a different radial stiffness behaviour of the stent. The numerical results show that composite polymer stents can improve the mechanical properties of the stent structure with their good radial behaviour, which meet the stringent requirements for biodegradable stents. The use of bioresorbable materials, which could reduce the risk of late in-stent thrombosis due to resorption of the scaffold, together with the ability of 3D printing such stents, hold significant potential for personalisation and offer the possibility of combining different materials in composite stent structures.

## 5. Conclusions

Sixteen novel composite stent structures were created from four layers of PLA and PCL. Finite element analysis was performed for all structures to provide insight into their mechanical performance during crimping and expansion. Stresses, plastic strains, foreshortening, and elastic recoil were evaluated. The following conclusions can be summarised from the analysis results:

- The structures with the same number of layers per material show similar stress, plastic strain, foreshortening and elastic recoil.
- The order of layers only impacts plastic strains for configurations with three PCL layers and a single PLA layer. In other configurations, the layer arrangement does not play a significant role.
- The volumetric ratio of elements has a more significant influence on stent foreshortening and elastic recoil than layer arrangement.
- A combination of different materials in a composite stent could be useful for customizing mechanical properties along the length of the stent. Matching the compliance of the stent and the blood vessel could lower the risk of short-term complications after stent deployment.

## Figures and Tables

**Table 1:** Stent dimensions.

Parameter	Symbol	Value
Outer stent diameter	*D*	4.4 mm
Inner stent diameter	*d*	4 mm
Total cell length	*L*	3.2 mm
Cell length	*A*	2.8 mm
Cell height	*c*	0.73 mm
Number of cells per circumference	*N*	8 mm
Strut width	*t*	0.2 mm
Stent thickness	*t_s_*	0.2 mm
Connecting strut width	*b*	0.3 mm
Radius 1	R_1_	0.2 mm
Radius 2	*R*_2_	0.41 mm

**Table 2:** Mechanical properties for materials considered in this study, adapted from [25,41].

Material	Density [kg/m^3^]	Poisson’s Ratio [–]	Young’s Modulus [MPa]	Yield Strength [MPa]	Tangent Modulus [MPa]	Material Model
PLA	1250	0.33	3000	65	30	Bilinear
PCL	1100	0.33	350	25	10	Bilinear

**Table 3:** Finite element mesh properties for numerical analysis.

Part	Element Type	Element Order	Number of Nodes	Number of Elements
Stent	Hex20 (SOLID186)	Quadratic	28,632	5120
Crimper	Quad4 (SHELL181)	Linear	25	16
Balloon	Quad4 (SHELL181)	Linear	168	140

**Table 4:** Equivalent plastic strain after final analysis step for different material configurations.

Configuration (0-PLA; 1-PCL)	Equivalent Plastic Strain [mm/mm]	Configuration (0-PLA; 1-PCL)	Equivalent Plastic Strain [mm/mm]
0000	0.72	1000	0.74
0001	0.72	1001	0.72
0010	0.71	1010	0.72
0011	0.71	1011	0.68
0100	0.72	1100	0.73
0101	0.71	1101	0.69
0110	0.71	1110	0.71
0111	0.65	1111	0.61

## Data Availability

The data presented in this study are available on request from the corresponding author.
